# Thioester-Containing Proteins in the *Drosophila melanogaster* Immune Response against the Pathogen *Photorhabdus*

**DOI:** 10.3390/insects11020085

**Published:** 2020-01-28

**Authors:** Ioannis Eleftherianos, Upasana Sachar

**Affiliations:** Infection and Innate Immunity Lab, Department of Biological Sciences, The George Washington University, Washington, DC 20052, USA; upasanashokal@gmail.com

**Keywords:** *Drosophila melanogaster*, thioester-containing proteins, *Photorhabdus*, *Heterorhabditis*, humoral immunity, cellular immunity, metabolism, infection

## Abstract

The fruit fly *Drosophila melanogaster* forms a magnificent model for interpreting conserved host innate immune signaling and functional processes in response to microbial assaults. In the broad research field of host-microbe interactions, model hosts are used in conjunction with a variety of pathogenic microorganisms to disentangle host immune system activities and microbial pathogenicity strategies. The pathogen *Photorhabdus* is considered an established model for analyzing bacterial virulence and symbiosis due to its unique life cycle that extends between two invertebrate hosts: an insect and a parasitic nematode. In recent years, particular focus has been given to the mechanistic participation of the *D. melanogaster* thioester-containing proteins (TEPs) in the overall immune capacity of the fly upon response against the pathogen *Photorhabdus* alone or in combination with its specific nematode vector *Heterorhabditis bacteriophora*. The original role of certain TEPs in the insect innate immune machinery was linked to the antibacterial and antiparasite reaction of the mosquito malaria vector *Anopheles gambiae*; however, revamped interest in the immune competence of these molecules has recently emerged from the *D. melanogaster*-*Photorhabdus* infection system. Here, we review the latest findings on this topic with the expectation that such information will refine our understanding of the evolutionary immune role of TEPs in host immune surveillance.

## 1. Introduction

Insects are excellent systems for analyzing the molecular and functional basis of host-pathogen interactions [1]. In particular, the fruit fly *Drosophila melanogaster* has played a crucial role in determining the signaling pathways and mechanisms they regulate in response to infection with a diverse range of microorganisms, including insect-specific pathogens as well as pathogens that infect humans. In the past several years, fine studies using genetic and genomic tools in *D. melanogaster* have uncovered evolutionarily conserved mechanisms of host defense [2,3,4]. These findings have significantly advanced the broad field of innate immunology and opened novel avenues of investigation into the identification and characterization of immune reactions directed against eukaryotic parasites [5]. Innate immune antimicrobial responses in *D. melanogaster* include systemic and tissue-specific humoral and cellular processes controlled by the Toll and Immune Deficiency (Imd) signaling pathways with the c-Jun-terminal kinase (Jnk) pathway and the Janus kinase (Jak)/signal transducer and activator of transcription (Stat) participating in complementary immune activities [6,7,8,9].

Entomopathogenic nematodes in the genus *Heterorhabditis* are widely used in crop protection practices for the management of a wide range of insect pests that mainly live in the soil. Their high efficiency is due to their ability to penetrate the insect cuticle and rapidly migrate in the host, their ability during infection to produce molecules that compromise the insect immune system, and their species-specific association with the Gram-negative bacteria *Photorhabdus* that synthesize and secrete a cocktail of virulence factors, which accelerate insect death by targeting mainly the gut and fat body as well as the hemocytes [10,11]. Because of these complex interactions involving two invertebrate animals and a microbe, entomopathogenic nematodes are also premier experimental tools for deciphering gene-for-gene interactions that define bacterial symbiosis and pathogenicity, nematode parasitism, and insect immunity [12]. The properties of this model have been recently adopted to dissect the insect host immune response against the nematode and their related bacteria when infected together or separately. These investigations have led to the determination of the genetic basis of host innate immune processes activated in response to the combined assault of the two pathogens or each one individually [13,14].

Here, we review the most recent information on the association of thioester-containing proteins (TEPs) in the interaction between *D. melanogaster* and the *Heterorhabditis* symbionts *Photorhabdus* and the impact of these molecules on the host response to these entomopathogenic bacteria. TEPs are critical factors for modulating innate immunity in *D. melanogaster* [15]. Accumulating knowledge on host-parasite relationships will lead to the discovery of novel nematode-bacterial strategies for targeting specific host immune-related components as well as host defense systems designed to oppose deadly attacks by entomopathogens.

## 2. Thioester-Containing Proteins in the *Drosophila* Humoral Response against *Photorhabdus*

The humoral arm of the *D. melanogaster* host defense is mostly based on the activation of microbial recognition receptors that induce intracellular signals regulated by evolutionarily conserved pathways, a process that ultimately leads to the synthesis and secretion of antimicrobial peptides in the open circulatory system of the fly [16,17]. However, the connection between *Tep* gene activity and regulation of humoral immune activity in *D. melanogaster* responding to entomopathogenic bacteria and their nematode vectors forms a recently explored research area in the broad field of insect immunology.

**Figure 1:**
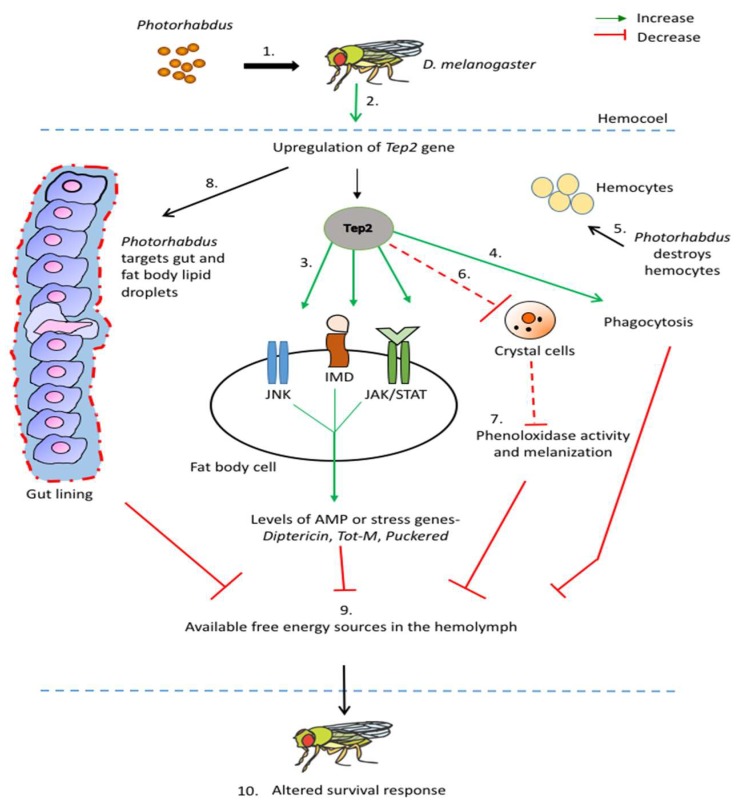
Model for the participation of TEP2 in the humoral and cellular immune response of *Drosophila melanogaster* wild-type adult flies against *Photorhabdus* entomopathogenic bacteria. 1. *Photorhabdus* enters the hemolymph of the flies. 2. *Tep2* is upregulated in fly tissues such as fat body and hemocytes after *Photorhabdus* injection. 3. Upregulation of *Tep2* results in the activation of Immune Deficiency (Imd), Janus kinase (Jak)/signal transducer and activator of transcription (Stat), and c-Jun-terminal kinase (Jnk) pathways leading to upregulation of antimicrobial peptide (AMP) and stress-related genes such as *Diptericin*, *Turandot-M* (Tot-M), and *Puckered*. 4. Upregulation of *Tep2* increases phagocytosis in response to *Photorhabdus* infection. 5. *Photorhabdus* targets and kills hemocytes (specifically plasmatocytes). 6. Upregulation of *Tep2* results in the appearance of fewer crystal cells in the hemolymph after *Photorhabdus* infection. 7. Reduced number of crystal cells leads to decreased phenoloxidase activity and melanization response. 8. *Photorhabdus* targets the gut (shown as red dashed border) and fat body lipid droplets promoting increased pathogen burden in the fly and consequently leading to increased cell death (activation of caspase Dronc). 9. The fly uses available energy sources (such as triglycerides and trehalose) to combat *Photorhabdus* infection. 10. The interaction between the *Drosophila* immune system activated through *Tep2* and *Photorhabdus* pathogenicity strategies results in altered survival of the fly. Solid red lines denote inhibition and dashed red lines denote decrease.

Recently, a series of studies analyzed immune signaling variation in flies deficient for certain *Tep* genes in the context of infection with the potent insect pathogenic bacterium *Photorhabdus* [18]. Because *Tep4* was upregulated in *D. melanogaster* adults in response to infection with either *Photorhabdus luminescens* or *Photorhabdus asymbiotica* bacteria and *Tep4* loss-of-function mutant flies were resistant to these pathogens, the investigators estimated the transcriptional expression of readout genes in *Photorhabdus*-infected *Tep4* mutant flies. The results demonstrated that two antimicrobial-peptide-encoding genes *Defensin* and *Cecropin-A1* were highly upregulated in the *Tep4* mutant flies in relation to the background controls when infected with either bacterial species, indicating the regulatory role of *Tep4* in the Toll and Imd signaling of *D. melanogaster* to *Photorhabdus*. In a similar fashion, it was further shown that *Tep6* mutants were resistant to both *Photorhabdus* species, while *Tep2* mutants were resistant to *P. asymbiotica* bacteria only, and that *Tep6* but not *Tep2* expression was required for the activation of Toll signaling, while *Tep2* only modulated Imd signaling [19]. In addition, Jak/Stat activity against either *Photorhabdus* species when *Tep2* is normally expressed and both *Tep2* and *Tep6* are needed for proper Jnk signaling in flies responding specifically to *P. asymbiotica* (Figure 1).

## 3. Thioester-Containing Proteins in the *Drosophila* Cellular Response against *Photorhabdus*

The cellular part of the innate immune response in *D. melanogaster* operates together with humoral factors to eliminate microbial intruders. Cellular immunity in *D. melanogaster* larvae and adult flies is controlled by the different types of hemocytes, which specialize in various immune activities that mainly include the detection, phagocytosis, and encapsulation of pathogens [20,21].

To identify the effect of TEP molecules on the *D. melanogaster* antibacterial cell-mediated immunity, recent works have focused on estimating the activity of cellular responses in flies with compromised *Tep* gene expression upon infection with potent entomopathogenic *Photorhabdus* bacteria. It was found that the presence of functional hemocytes in adult *D. melanogaster* is essential for the expression of *Tep2* and *Tep6* during infection with *P. luminescens* or *P. asymbiotica* bacteria, as functional inactivation of hemocytes in flies leads to decreased expression of these two genes in the presence of either pathogen [19]. *Tep2* and *Tep6* gene activity in *D. melanogaster* also impacts hemocyte activation and viability against *Photorhabdus* because total hemocyte numbers are altered in flies with mutations in either *Tep2* or *Tep6*, and these mutants contain a larger proportion of live hemocytes than control individuals when the pathogenic bacteria are present in the hemolymph (Figure 1). These changes are accompanied by modification in phagocytosis activity in the *Tep* mutant flies, as indicated by their reduced ability to engulf *Escherichia coli* inactive opsonized bioparticles and the differential expression of the phagocytic receptor Eater in response to *Photorhabdus* challenge [19]. In these experiments, *E. coli* particles were used because *Photorhabdus* pathogens are able to interfere with insect phagocytosis [14]. In another more detailed work, *D. melanogaster* TEP4 was also assigned a significant role in the regulation of cellular immune mechanisms against *P. luminescens* or *P. asymbiotica* bacteria [22]. Specifically, in flies compromised by these pathogenic bacteria, the presence of functional hemocytes is critical for the transcriptional expression of *Tep4* at normal levels in wild-type flies as well as for the survival of *Tep4* loss-of-function mutants. In addition, interfering with the expression of *Tep4* in adult flies reduces total hemocyte numbers in the hemolymph, an effect that is accompanied by simultaneous increase in the proportion of crystal cells and changes in phagocytic ability against *Photorhabdus*.

The phenoloxidase and melanization reactions in *D. melanogaster* bridge the humoral and cellular arms of the innate antimicrobial immune response [23,24]. In a curious way, certain TEPs are also capable of modulating these two responses in the fly upon infection with entomopathogenic bacteria. In the absence of *Tep2* or *Tep4*, but not *Tep6*, both phenoloxidase activity in the hemolymph and melanization intensity at the injection site increase substantially in the mutants when responding to *Photorhabdus* infection (Figure 1) [18,19].

## 4. Thioester-Containing Proteins and *Drosophila* Metabolism during *Photorhabdus* Infection

An appealing question in insect immunology is the determination of the host factors that link immunity and metabolic function in the context of microbial infection. The close relationship between immune regulation and metabolic homeostasis in health and disease is well established [25,26,27,28], and evidence on the host signaling pathways and their molecules linking these important biological processes has recently started to emerge due to studies in the *D. melanogaster* model [29,30,31]. Because certain TEPs in *D. melanogaster* were shown to play a regulatory role by inducing humoral and cellular immune activities against *Photorhabdus* pathogens [18,19,22], these molecules also form a reliable indicator for their potential multipurpose involvement in linking host immunity and metabolism in the presence of pathogenic bacteria. Of note, injection of *P. luminescens* into the hemocoel of background wild-type flies upregulates both *Tep2* and *Tep4* in the fat body as well as Tep4 in the gut, whereas injection of *P. asymbiotica* increases the expression of *Tep4* in the fat body only (Figure 1) [32]. Although the tissue-specific expression pattern of *Tep* genes in *D. melanogaster* varies upon infection with different bacterial pathogens [33], these results demonstrate that certain *Tep* genes are expressed in the two metabolic tissues of the fly—fat body and gut—in response to *Photorhabdus* systemic infection.

To address these issues, a recent study monitored the metabolic activity in *D. melanogaster* TEP-deficient flies during response to infection by two *Photorhabdus* species as well as by a nonpathogenic strain of *E. coli* used as control and estimated the amounts of different metabolites including carbohydrates and lipids [32]. Results from these experiments first revealed that trehalose levels are drastically affected in flies with compromised *Tep2*, regardless of whether they are infected with pathogenic *Photorhabdus* bacteria or nonpathogenic *E. coli*. Examination of glycogen levels similarly indicated that *Tep4* gene activity is critical for the utilization of glycogen during the course of pathogenic or nonpathogenic challenge. Interestingly, glucose levels in infected *D. melanogaster* flies are differentially affected depending on the impairment of distinct *Tep* genes. More precisely, infection of *Tep2* mutants with *P. asymbiotica*, but not *P. luminescens*, increases free glucose levels, while infection of *Tep4*-defective adults with either *P. asymbiotica* pathogens or nonpathogenic *E. coli* boosts glucose concentration in the infected flies. Also, both *Tep2* and *Tep4* gene activities in *D. melanogaster* adults seem to interfere with the regulation of triglyceride levels following *Photorhabdus* inoculation, since suppression of either *Tep2* or *Tep4* gene expression elevates the amount of triglycerides upon infection of the *Tep* mutant flies with either *P. luminescens* or *P. asymbiotica* compared with controls. Similarly, examination of fat body morphology in infected and uninfected *D. melanogaster* shows that *Tep2*- and *Tep4*-inactivated flies contain bigger-sized lipid droplets than infected wild-type individuals, an effect that is observable only during infection with pathogenic *Photorhabdus* bacteria but not in uninfected flies or those injected with *E. coli*. These results indicate that the absence of TEP2 or TEP4 may facilitate metabolic activity upon *Photorhabdus* infection. Overall, these findings have uncovered a previously unknown correlation between *Tep* gene expression and metabolic activity in *D. melanogaster* wild-type flies responding to the pathogen *Photorhabdus*. Future work is expected to resolve the exact involvement of TEP molecules in the *D. melanogaster* immunometabolic response to *Photorhabdus* infection.

## 5. Thioester-Containing Protein Gene Expression in *Drosophila* and Stress and Inflammation Response to *Photorhabdus*

Variations in insect survival and pathogen load often correlate with changes in host innate immune response, which is often difficult to separate from stress or inflammatory responses to a given microbial challenge [34,35]. A recent study expanded on this topic by examining the contribution of *Tep* gene activity to the stress and inflammation response to infection by *Photorhabdus* bacteria [32]. Because the nitrite level (a by-product of nitric oxide synthesis) is an indicator for stress response in the fly [36], the authors estimated nitrite amounts produced in *D. melanogaster* adults compromised for either *Tep2* or *Tep4* in the context of infection with either *P. luminescens* or *P. asymbiotica*. The results from these experiments indicated that inactivation of either *Tep* gene leads to higher concentration of nitrite in the mutant flies infected with these pathogens. This effect is particularly pronounced during the late phase of the infection, which coincides with the spread of the bacteria in the fly hemocoel and colonization of the gut and fat body tissues [37]. In addition, *Tep2* and *Tep4* loss-of-function mutant flies injected with *Photorhabdus* bacteria exhibited lower expression of the caspase-encoding *Dronc*, which pointed towards decreased levels of apoptosis [38]. These findings were confirmed at the tissue level by detecting lower levels of protein expression of death caspase-1 in the midgut of *Tep2* and *Tep4 Photorhabdus*-infected flies [39]. The latter study provides evidence for the role of *Tep* gene activity in modulating cell death during infection with *Photorhabdus* pathogenic bacteria. Whether this effect is due to the higher numbers of *Photorhabdus* in the infected tissues due to the absence of apoptosis or the decreased release of the bacteria from the infected host cells that in turn might reduce the capacity of the pathogens to spread in the *Tep* mutant flies is currently unknown and therefore is worth investigating in future studies.

## 6. Role of Thioester-Containing Proteins in the *Drosophila* Antinematode Response

Recent transcriptomic studies in *D. melanogaster* have implicated the role of *Tep* genes in the interaction of parasitic nematode infection with the adult fly and larval innate immune response. Microarray analysis of the differentially regulated genes in *D. melanogaster* larvae responding to symbiotic *Heterorhabditis bacteriophora* identified *Tep2* among the 100 most enriched genes as well as *Tep1*, and the C3 homolog *Tep3* and *Tep4* were also induced by infection with these nematode parasites [40]. Analysis of the survival response of *Tep* loss-of-function mutant larvae upon challenge with *H. bacteriophora* symbiotic nematodes revealed increased sensitivity of individuals with defective *Tep3* only or when together with *Tep2*, while deficiencies in *Tep2* or *Tep4* alone failed to differentiate larval survival ability compared to background controls. Although further functional analysis was not attempted in this study, the obtained findings indicate that certain TEP molecules play an as-yet unidentified direct or indirect role in regulating the antinematode immune reaction [40]. Similar to the research with *Photorhabdus* bacteria, the functional basis of this reaction might be linked to the capacity of TEPs (TEP3 in this case and probably TEP2) to enhance the activity of humoral immune components or influence the stimulation and efficacy of immune cells to recognize and entrap parasitic nematodes. Interestingly, a subsequent RNA-sequencing study involving exposure of *D. melanogaster* adults with either symbiotic (carrying *P. luminescens* bacteria) or axenic (lacking *P. luminescens*) nematodes of *H. bacteriophora* showed that either type of infection results in consistent downregulation of *Tep1* at early and late times after the introduction of the pathogens to the flies, while *Tep1* and *Tep2* together with *Tep4* are highly upregulated by the parasites as well as by injection of *P. luminescens* bacteria alone throughout the infection [41]. Similar to the work with *H. bacteriophora*, infection of *D. melanogaster* larvae with the entomopathogenic nematodes *Steinernema carpocapsae* containing their symbiotic *Xenorhabdus nematophila* bacteria leads to high levels of induction for *Tep1* and *Tep2*, which remains steady during the infection [42]. Therefore, the challenge awaiting in the future involves the identification and mechanistic characterization of each of these transcriptomically detected TEPs in *D. melanogaster*, or more broadly, insect antinematode innate immune action.

## 7. Conclusions

The insect immune system includes extracellular and intracellular signaling pathways, which provide an efficient and rapid response against a variety of microbial pathogens that constantly employ novel tactics to undermine the host defense. Insect TEPs belong to a selective group of molecules with evolutionarily conserved function to first recognize the presence of microbial intruders and subsequently activate diverse immune mechanisms that restrict microbial growth and facilitate microbial elimination from the host [43]. In *D. melanogaster*, recent research employing the model pathogenic bacterium *Photorhabdus* and its parasitic nematode vector *Heterorhabditis* has assigned a putative role as immune regulators to TEP2, TEP4, and TEP6, which are markedly induced in response to *Photorhabdus* and evidently possess the capacity to control humoral and cellular reactions during infection with the mutualistic bacteria alone or the nematode-bacteria complexes. Changes in TEP transcriptional activity in *D. melanogaster* adult flies substantially modify the potency of immune activities including variation in innate immune signaling and therefore expression of antimicrobial peptide and stress-related genes as well as a decline in phenoloxidase and melanization responses. These drastic changes in turn alter the survival ability of the flies upon challenge with the pathogens. These findings in the *D. melanogaster* model have transformed our current knowledge of the existence of factors that are able to adjust the insect host innate immune system to oppose specific microbial invaders. Future focus should be given to analyzing the tissue-specific expression of *Tep* genes in *D. melanogaster* responding to nematode infection and determine the functional involvement of each TEP molecule in the fly antinematode defense. Such research will aim at refining further the details of these mechanisms with the prospect that the outcome will lead to more efficient strategies for the management of deleterious crop pests and vectors of deadly diseases.

## References

[1] Sackton T.B. (2019). Comparative genomics and transcriptomics of host-pathogen interactions in insects: Evolutionary insights and future directions. Curr. Opin. Insect Sci..

[2] Buchon N., Silverman N., Cherry S. (2014). Immunity in *Drosophila melanogaster*—From microbial recognition to whole-organism physiology. Nat. Rev. Immunol..

[3] Gold K.S., Brückner K. (2015). Macrophages and cellular immunity in *Drosophila melanogaster*. Semin. Immunol..

[4] Parsons B., Foley E. (2016). Cellular immune defenses of *Drosophila melanogaster*. Dev. Comp. Immunol..

[5] Keebaugh E.S., Schlenke T.A. (2014). Insights from natural host-parasite interactions: The *Drosophila* model. Dev. Comp. Immunol..

[6] Lindsay S.A., Wasserman S.A. (2014). Conventional and non-conventional *Drosophila* Toll signaling. Dev. Comp. Immunol..

[7] Zhai Z., Huang X., Yin Y. (2018). Beyond immunity: The Imd pathway as a coordinator of host defense, organismal physiology and behavior. Dev. Comp. Immunol..

[8] Myllymäki H., Rämet M. (2014). JAK/STAT pathway in *Drosophila* immunity. Scand. J. Immunol..

[9] Delaney J.R., Stöven S., Uvell H., Anderson K.V., Engström Y., Mlodzik M. (2006). Cooperative control of *Drosophila* immune responses by the JNK and NF-kappaB signaling pathways. EMBO J..

[10] Brivio M.F., Mastore M. (2018). Nematobacterial Complexes and Insect Hosts: Different Weapons for the Same War. Insects.

[11] Cooper D., Eleftherianos I. (2016). Parasitic Nematode Immunomodulatory Strategies: Recent Advances and Perspectives. Pathogens.

[12] Shi Y.M., Bode H.B. (2018). Chemical language and warfare of bacterial natural products in bacteria-nematode-insect interactions. Nat. Prod. Rep..

[13] Castillo J.C., Reynolds S.E., Eleftherianos I. (2011). Insect immune responses to nematode parasites. Trends Parasitol..

[14] Eleftherianos I., ffrench-Constant R.H., Clarke D.J., Dowling A.J., Reynolds S.E. (2010). Dissecting the immune response to the entomopathogen *Photorhabdus*. Trends Microbiol..

[15] Lu Y., Su F., Li Q., Zhang J., Li Y., Tang T., Hu Q., Yu X.Q. (2020). Pattern recognition receptors in *Drosophila* immune responses. Dev. Comp. Immunol..

[16] Ganesan S., Aggarwal K., Paquette N., Silverman N. (2011). NF-kappaB/Rel proteins and the humoral immune responses of *Drosophila melanogaster*. Curr. Top. Microbiol. Immunol..

[17] Imler J.L., Bulet P. (2005). Antimicrobial peptides in *Drosophila*: Structures, activities and gene regulation. Chem. Immunol. Allergy.

[18] Shokal U., Eleftherianos I. (2017). Thioester-Containing Protein-4 Regulates the *Drosophila* Immune Signaling and Function against the Pathogen *Photorhabdus*. J. Innate Immun..

[19] Shokal U., Kopydlowski H., Eleftherianos I. (2017). The distinct function of Tep2 and Tep6 in the immune defense of *Drosophila melanogaster* against the pathogen *Photorhabdus*. Virulence.

[20] Wood W., Martin P. (2017). Macrophage Functions in Tissue Patterning and Disease: New Insights from the Fly. Dev. Cell.

[21] Honti V., Csordás G., Kurucz É., Márkus R., Andó I. (2014). The cell-mediated immunity of *Drosophila melanogaster*: Hemocyte lineages, immune compartments, microanatomy and regulation. Dev. Comp. Immunol..

[22] Shokal U., Eleftherianos I. (2017). The *Drosophila* Thioester-Containing Protein-4 participates in the induction of the cellular immune response to the pathogen *Photorhabdus*. Dev. Comp. Immunol..

[23] Tang H. (2009). Regulation and function of the melanization reaction in *Drosophila*. Fly.

[24] Eleftherianos I., Revenis C. (2011). Role and importance of phenoloxidase in insect hemostasis. J. Innate Immun..

[25] Ganeshan K., Chawla A. (2014). Metabolic regulation of immune responses. Annu. Rev. Immunol..

[26] Vavricka C.J., Han Q., Mehere P., Ding H., Christensen B.M., Li J. (2014). Tyrosine metabolic enzymes from insects and mammals: a comparative perspective. Insect Sci..

[27] Brestoff J.R., Artis D. (2015). Immune regulation of metabolic homeostasis in health and disease. Cell.

[28] Zmora N., Bashiardes S., Levy M., Elinav E. (2017). The Role of the Immune System in Metabolic Health and Disease. Cell Metab..

[29] Dionne M. (2014). Immune-metabolic interaction in *Drosophila*. Fly.

[30] Lee K.A., Lee W.J. (2018). Immune-metabolic interactions during systemic and enteric infection in *Drosophila*. Curr. Opin. Insect Sci..

[31] Galenza A., Foley E. (2019). Immunometabolism: Insights from the *Drosophila* model. Dev. Comp. Immunol..

[32] Shokal U., Kopydlowski H., Harsh S., Eleftherianos I. (2018). Thioester-Containing Proteins 2 and 4 Affect the Metabolic Activity and Inflammation Response in *Drosophila*. Infect. Immun..

[33] Bou Aoun R., Hetru C., Troxler L., Doucet D., Ferrandon D., Matt N. (2011). Analysis of thioester-containing proteins during the innate immune response of *Drosophila melanogaster*. J. Innate Immun..

[34] Davies S.A., Overend G., Sebastian S., Cundall M., Cabrero P., Dow J.A., Terhzaz S. (2012). Immune and stress response ‘cross-talk’ in the *Drosophila* Malpighian tubule. J. Insect Physiol..

[35] Asri R.M., Salim E., Nainu F., Hori A., Kuraishi T. (2019). Sterile induction of innate immunity in *Drosophila melanogaster*. Front. Biosci..

[36] Dow J.A., Romero M.F. (2010). *Drosophila* provides rapid modeling of renal development, function and disease. Am. J. Physiol. Renal Physiol..

[37] Nielsen-LeRoux C., Gaudriault S., Ramarao N., Lereclus D., Givaudan A. (2012). How the insect pathogen bacteria *Bacillus thuringiensis* and *Xenorhabdus*/*Photorhabdus* occupy their hosts. Curr. Opin. Microbiol..

[38] Ming M., Obata F., Kuranaga E., Miura M. (2014). Persephone/Spätzle pathogen sensors mediate the activation of Toll receptor signaling in response to endogenous danger signals in apoptosis-deficient *Drosophila*. J. Biol. Chem..

[39] Song Z., McCall K., Steller H. (1997). DCP-1, a *Drosophila* cell death protease essential for development. Science.

[40] Arefin B., Kucerova L., Dobes P., Markus R., Strnad H., Wang Z., Hyrsl P., Zurovec M., Theopold U. (2014). Genome-wide transcriptional analysis of *Drosophila* larvae infected by entomopathogenic nematodes shows involvement of complement, recognition and extracellular matrix proteins. J. Innate Immun..

[41] Castillo J.C., Creasy T., Kumari P., Shetty A., Shokal U., Tallon L.J., Eleftherianos I. (2015). *Drosophila* anti-nematode and antibacterial immune regulators revealed by RNA-seq. BMC Genom..

[42] Yadav S., Daugherty S., Shetty A.C., Eleftherianos I. (2017). RNAseq analysis of the *Drosophila* response to the entomopathogenic nematode *Steinernema*. G3 (Bethesda).

[43] Shokal U., Eleftherianos I. (2017). Evolution and Function of Thioester-Containing Proteins and the Complement System in the Innate Immune Response. Front. Immunol..

